# Identification of sodium channel isoforms that mediate action potential firing in lamina I/II spinal cord neurons

**DOI:** 10.1186/1744-8069-7-67

**Published:** 2011-09-12

**Authors:** Michael E Hildebrand, Janette Mezeyova, Paula L Smith, Michael W Salter, Elizabeth Tringham, Terrance P Snutch

**Affiliations:** 1 Zalicus Pharmaceuticals Ltd., Vancouver, BC, Canada; 2 Program in Neurosciences & Mental Health, Hospital for Sick Children, Toronto, ON, Canada; 3 Department of Physiology, University of Toronto, Toronto, ON, Canada; 4 Michael Smith Laboratories, University of British Columbia, Vancouver, BC, Canada

**Keywords:** sodium channel, Na_V_1.2, Na_V_1.3, nociception, lamina I/II, dorsal horn, spinal cord, entire soma isolation

## Abstract

**Background:**

Voltage-gated sodium channels play key roles in acute and chronic pain processing. The molecular, biophysical, and pharmacological properties of sodium channel currents have been extensively studied for peripheral nociceptors while the properties of sodium channel currents in dorsal horn spinal cord neurons remain incompletely understood. Thus far, investigations into the roles of sodium channel function in nociceptive signaling have primarily focused on recombinant channels or peripheral nociceptors. Here, we utilize recordings from lamina I/II neurons withdrawn from the surface of spinal cord slices to systematically determine the functional properties of sodium channels expressed within the superficial dorsal horn.

**Results:**

Sodium channel currents within lamina I/II neurons exhibited relatively hyperpolarized voltage-dependent properties and fast kinetics of both inactivation and recovery from inactivation, enabling small changes in neuronal membrane potentials to have large effects on intrinsic excitability. By combining biophysical and pharmacological channel properties with quantitative real-time PCR results, we demonstrate that functional sodium channel currents within lamina I/II neurons are predominantly composed of the Na_V_1.2 and Na_V_1.3 isoforms.

**Conclusions:**

Overall, lamina I/II neurons express a unique combination of functional sodium channels that are highly divergent from the sodium channel isoforms found within peripheral nociceptors, creating potentially complementary or distinct ion channel targets for future pain therapeutics.

## Background

Voltage-gated sodium channels play critical roles in regulating neuronal excitability throughout the nervous system. Along with other types of voltage-gated ion channels, they contribute to the initiation, generation, and propagation of action potentials and can also modulate excitability via subthreshold conductances. Sodium channels are composed of a pore-forming α subunit and may also contain accessory (β) subunits that alter channel properties. The nine different sodium channel α subunit isoforms identified, termed Na_V_1.x, display heterogeneity in distribution, expression and function, yet all are greater than 50% identical in amino acid sequence in their transmembrane and extracellular domains [1]. Of the nine isoforms, Na_V_1.5, Na_V_1.8, and Na_V_1.9 are termed tetrodotoxin (TTX)-resistant as they are insensitive to nanomolar concentrations of TTX [1].

To date, four of the sodium channel isoforms have been implicated in nociceptive signaling mechanisms. Genetic knockout and/or antisense knockdown of the Na_V_1.3, Na_V_1.7, Na_V_1.8, and Na_V_1.9 channels results in attenuation of acute and/or chronic pain responses in rat and mouse models (for example, see [2]). The distribution and relative expression of these pronociceptive sodium channel isoforms are also altered during chronic inflammatory and neuropathic pain states [3-5]. Furthermore, both loss-of-function and gain-of-function mutations in Na_V_1.7 have been linked to inherited human pain disorders. A number of anticonvulsant and antiarrhythmic therapeutics that were developed using target-blind, traditional pharmacological approaches are now used for the treatment of neuropathic pain (e.g., lacosamide, lamotrigine, mexilitine) and have been shown to inhibit multiple sodium channel isoforms in a state-dependent manner. At present, considerable effort is being invested into developing therapeutic compounds that selectively target individual pronociceptive sodium channel isoforms [6,7].

The role of the Na_V_1.3 sodium channel isoform in neuropathic pain remains somewhat controversial. Antisense knockdown of Na_V_1.3 has been shown to either attenuate [8,9] or have no effect [10] on behavioral hypersensitivity in rat models of peripheral and central neuropathic pain, while Na_V_1.3 knockout mice show normal pain behavior [11]. However, the study of Na_V_1.3 channel function has been limited by a combination of factors: within adult dorsal root ganglia (DRG) neurons Na_V_1.3 channels are only significantly expressed after the induction of neuropathic pain states, and these DRG neurons also express the pronociceptive TTX-sensitive Na_V_1.7 isoform [3]. Although Na_V_1.3 protein upregulation has been linked to hyperexcitable responses within nociceptive dorsal horn spinal cord neurons following induction of either peripheral [9] or central neuropathic pain [8], the specific changes in sodium channel properties within these neurons are unknown. Despite strong clinical evidence for sodium channel function in neuropathic pain states and the need to understand the roles of specific sodium channel isoforms in all components of the nociceptive pathway, the study of nociceptive sodium channels has been almost exclusively limited to heterologous systems and peripheral nociceptors (DRG neurons).

Neurons in superficial layers of the dorsal horn (lamina I and lamina II) integrate and relay acute and chronic nociceptive signals from peripheral nociceptors to pain processing regions in the brain. Plasticity at peripheral nociceptor - lamina I/II neuron synapses [12] as well as changes in the intrinsic excitability of lamina I/II neurons [8] have been linked to central sensitization mechanisms underlying chronic inflammatory and neuropathic pain [13,14]. Using an *in situ *spinal cord slice preparation, voltage clamp recordings of sodium channel currents from intact lamina I/II neurons are virtually impossible to resolve, largely due to space clamp challenges that are compounded by extremely fast channel kinetics and high channel expression in distal axons and terminals [15]. To address these challenges, Safronov and colleagues developed a novel recording configuration whereby the soma (and potentially a proximal process) of a dorsal horn neuron is pulled off of the spinal cord slice surface through slow withdrawal of the recording pipette (termed entire soma isolation, ESI) [16,17]. However, the functional properties of voltage-gated sodium channels in lamina I/II neurons remain to be comprehensively characterized.

We recently reported the design and synthesis of a novel small organic compound (Z123212) that potently reduced the excitability of lamina I/II spinal cord neurons and reversed behavioral responses in animal models of acute and chronic pain [18]. We predict that, at least in part, the analgesic effects of Z123212 are a result of its ability to selectively stabilize the slow inactivated state of sodium channels expressed in lamina I/II neurons [18]. In this regard, voltage-gated sodium channels localized within dorsal horn neurons may represent a novel therapeutic pain target. In this study we combine electrophysiological and pharmacological experiments with quantitative real-time RT-PCR (qRT-PCR) to systematically investigate sodium channel isoforms functionally expressed within lamina I/II spinal cord neurons and to explore the physiological implications resulting from these findings.

## Results

### Voltage-Gated Sodium Channel Currents from Lamina I/II Neurons are TTX-Sensitive

To characterize the functional and pharmacological properties of sodium channel currents within lamina I/II neurons, we performed voltage clamp recordings on lamina I/II neurons from P6 to P9 Wistar rats, an age range amenable to the ESI recording configuration [16]. The ESI technique differs from the nucleated patch technique [19] in that the *entire *soma of a neuron sitting directly on the slice surface is withdrawn and isolated with or without the presence of a proximal process. In this configuration a sizeable fraction of the original sodium channel currents remained (I_peak _= -290 +/- 33 pA, n = 39) under conditions that created good space clamp, as illustrated by smooth current kinetics and a current-voltage relationship with appropriate slope factor (see below). The observation of peak sodium currents ranging from 100 pA (cutoff amplitude) to 950 pA indicated that the presence and length of proximal axon was variable [16]; however, none of the biophysical or pharmacological parameters tested varied with current amplitude.

**Figure 1:**
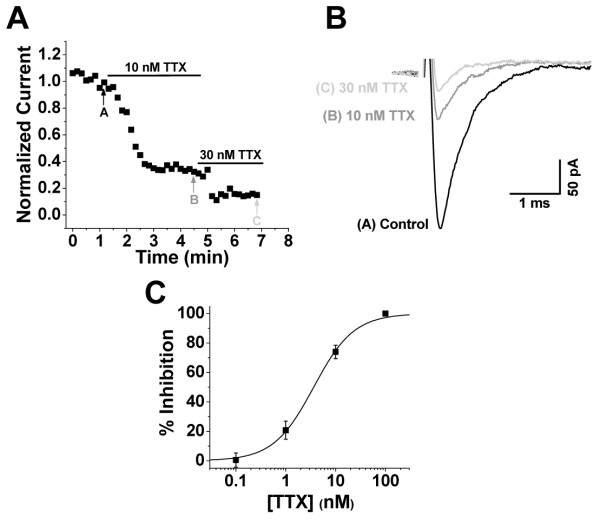
**Sodium channel currents in lamina I/II neurons are potently and completely blocked by TTX**. Voltage clamp recordings of sodium channel currents using the ESI technique on lamina I/II neurons from P6 to P9 Wistar rats. **A) **Representative time course of normalized peak sodium channel currents during depolarizations to -20 mV elicited every 10 seconds. Perfusion of 10 nM and 30 nM TTX caused a robust inhibition of sodium channel currents. **B) **Representative sodium channel current traces from specific time points (as labeled) in the time course shown in **A**. **C) **Concentration-response relationship for TTX. n = 3 to 5 for all data points. LJP = 4.2 mV, uncorrected.

Sodium channel currents within isolated lamina I/II neurons have previously been shown to be blocked by TTX, although these effects were not quantified to determine the potency and variability of blockade [16]. Examining concentration-dependent responses, we found that TTX concentrations of 100 nM and greater caused a complete (100%, n = 3) elimination of sodium channel currents in lamina I/II neurons (Figure 1). Thus, sodium channels functionally expressed within the soma and proximal processes of lamina I/II neurons are entirely TTX-sensitive. Furthermore, the inhibition of sodium currents by TTX was highly potent, with an IC_50 _= 3.6 nM and a slope factor = 1.08 (Figure 1), which completely aligns with the reported TTX IC_50 _values for recombinant TTX-sensitive sodium channels [1]. The high precision of the concentration-dependent response data (Figure 1C) demonstrates the pharmacological advantages of the ESI recording configuration over experiments on intact neurons from tissue slices, where drug access issues can both decrease potency and increase the variability of drug effects [20].

### Biophysical Properties of Sodium Channel Currents Within Lamina I/II Neurons

**Figure 2:**
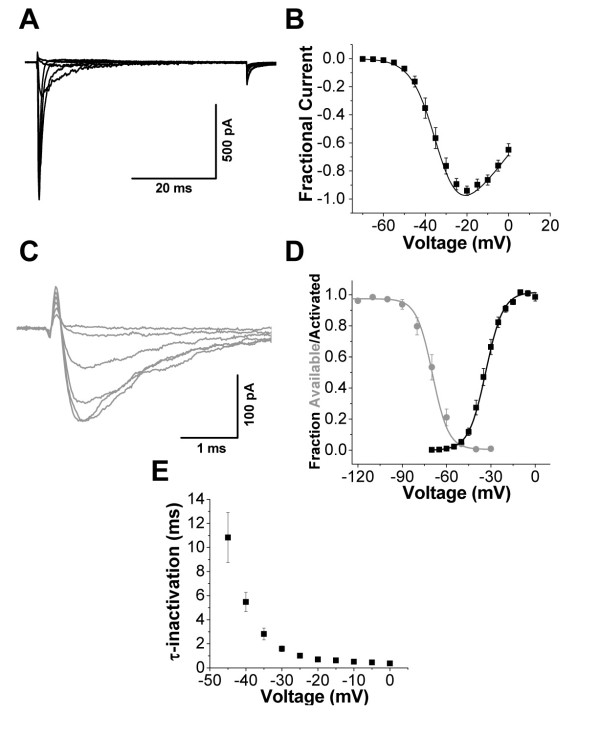
**Biophysical properties of sodium channel currents in lamina I/II neurons**. **A) **Voltage dependence of activation. Representative sodium channel current traces during depolarizing steps ranging between -60 mV and -10 mV at 10 mV increments. **B) **Average normalized peak current-voltage relationship for sodium channel current recordings as illustrated in **A**. **C) **Voltage dependence of fast inactivation. Representative sodium channel current traces elicited by steps to -20 mV preceded by 100 ms conditioning pulses between -90 and -40 mV at 10 mV increments. V_hold _= -100 mV. **D) **Steady-state activation (black; derived from **B**) and inactivation (grey) curves. **E) **Exponential fits of inactivation kinetics from sodium channel currents as in **A **demonstrate a steep voltage dependence of τ_inact_. n = 13 for all averaged data points. LJP = 4.2 mV, uncorrected.

We next investigated the functional characteristics of voltage-gated sodium channels expressed within lamina I/II neurons. Lamina I/II neuron sodium channel currents displayed a relatively hyperpolarized voltage dependence of activation (V_0.5Activation _= -34 +/- 1 mV, n = 13 and k_a _= 5.0 +/- 0.3 mV, n = 13; Figure 2A,B). Similarly, the voltage dependence of fast inactivation was relatively hyperpolarized (V_0.5FastInactivation _= -69 +/- 2 mV, n = 13 and k_i _= 5.5 +/- 0.3 mV, n = 13; Figure 2C,D). The potential physiological consequences of the observed overlap in activation and availability curves near resting membrane potential values (Figure 2D) was investigated in subsequent experiments. As observed for many recombinant TTX-sensitive sodium channel isoforms [1], fast inactivation of lamina I/II sodium channel currents exhibited rapid kinetics and a steep voltage dependence (Figure 2E).

**Figure 3:**
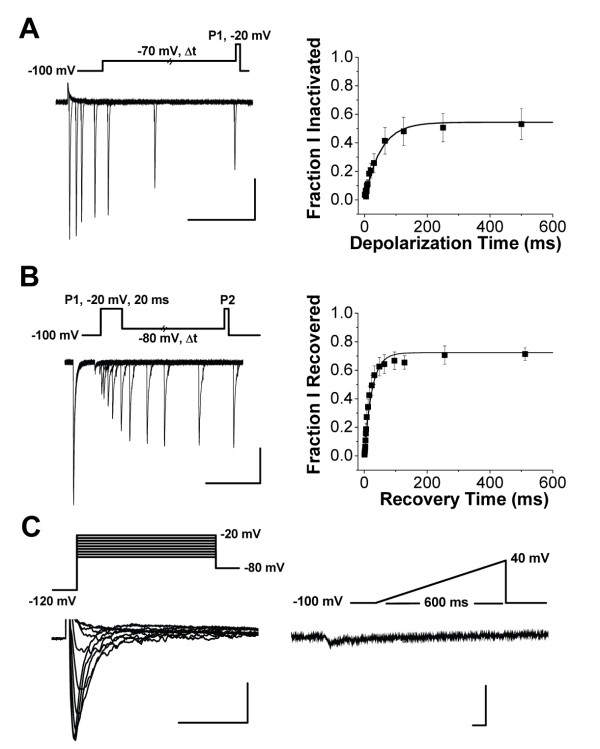
**Fast inactivation properties of sodium channel currents in lamina I/II neurons**. Voltage protocols are shown throughout for corresponding recording traces. For visual clarity, only a selected time period from a subset of recording sweeps are shown. **A) **Onset of closed-state inactivation. *Left*, representative sodium channel current traces following conditioning steps of variable length to -70 mV. Scale bar x axis = 50 ms, y axis = 100 pA. *Right*, fraction of sodium channel current that becomes inactivated for a given sweep normalized to peak current in the first sweep (n = 9). V_hold _= -100 mV. **B) **Recovery from fast inactivation. *Left*, representative sodium channel current traces. Scale bar x axis = 50 ms, y axis = 100 pA. *Right*, average fraction of P2 sodium channel current relative to P1 current amplitude (n = 10). V_hold _= -100 mV. **C) **Sodium channel currents elicited during ramp depolarizations (*right; *V_hold _= -100 mV) are only a small fraction of the amplitude of peak currents produced during a standard IV protocol with depolarizations between -60 mV and -20 mV (*left*). Scale bars x axes = 5 ms, y axes = 100 pA. LJP = 4.2 mV, uncorrected.

The complex inactivation properties of sodium channels partially determine how they will influence action potential firing threshold and frequency [21]. Within lamina I/II neurons, sodium channel currents displayed a relatively fast rate for the onset of closed-state inactivation (τ_onset _= 51 +/- 6 ms, n = 9 at -70 mV; Figure 3A). The sodium channels in lamina I/II neurons also rapidly recovered from inactivation (τ_recov _= 27 +/- 9 ms, n = 10 during recovery to -80 mV; Figure 3B). Currents did not completely recover at -80 mV, as a fraction of the current remained inactivated at this potential (see Figure 2D). Due to the fast rate of both closed state and open state inactivation, a 600 ms ramp depolarization from -100 mV to +40 mV elicited sodium channel currents that were only 6.6 +/- 1.0% (n = 7) of peak currents measured from a standard IV protocol (Figure 3C).

**Figure 4:**
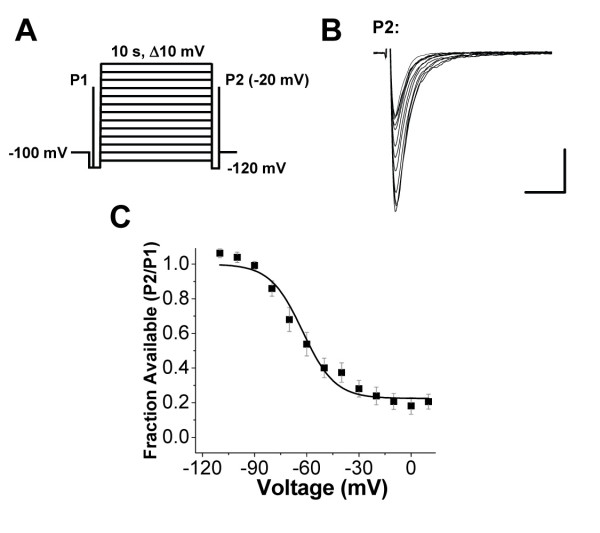
**Voltage dependence of sodium channel slow inactivation in lamina I/II neurons**. **A) **Voltage waveform protocol whereby P2 test currents follow a 10 second conditioning pulse at variable potentials. Currents were recovered from fast inactivation at -120 mV for 100 ms before P2. V_hold _= -100 mV. **B) **Representative sodium channel current traces during P2 tests pulses following 10 s conditioning pulses from -110 mV to + 10 mV. Scale bar x axis = 2 ms, y axis = 250 pA. **C) **Average fraction of P2 sodium channel current relative to P1 current amplitude during recordings as illustrated in A (n = 9). LJP = 4.2 mV, uncorrected.

The slow inactivated state of sodium channels appears to be advantageously targeted by some analgesics [18,22], so we measured the voltage dependence of sodium channel slow inactivation in lamina I/II neurons. Compared to fast inactivation, the slow inactivation-voltage curve had a slightly depolarized midpoint and a much shallower dependence on voltage (V_0.5SlowInactivation _= -62 +/- 3 mV, n = 9 and k_i _= 10 +/- 1 mV, n = 9; Figure 4).

### Lamina I/II Sodium Channels are Primarily Composed of Na_V_1.2 and Na_V_1.3 Isoforms

**Figure 5:**
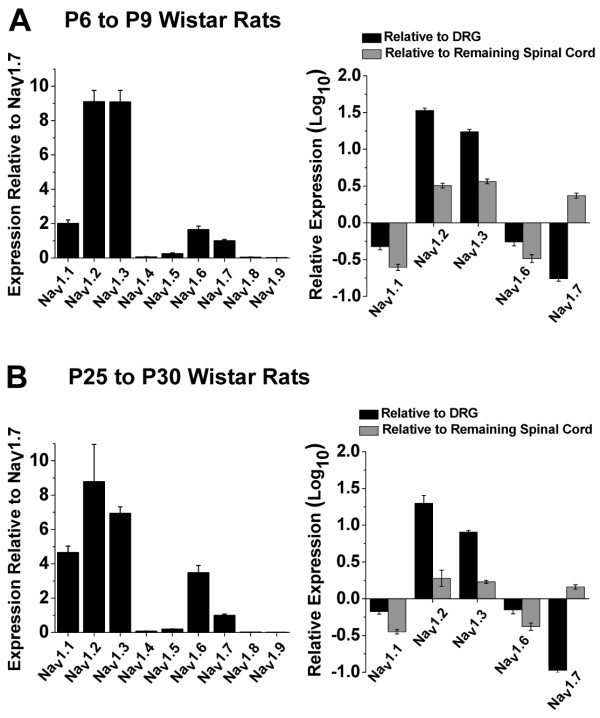
**Relative expression of sodium channel isoform mRNA in lamina I/II lumbar spinal cord**. qRT-PCR was performed on superficial horizontal dorsal horn slices of Wistar rats using sodium channel isoform-specific primers and normalization to an endogenous control (actin). **A) ***Left*, comparisons between sodium channel isoform expression relative to Na_V_1.7 revealed predominant expression of Na_V_1.2 and Na_V_1.3 isoforms in P6 to P9 rats (n = 5). *Right*, relative expression for the sodium channel isoforms that are robustly expressed within lamina I/II tissue compared to isoform expression within DRG tissue or lamina III to X spinal cord tissue. Note the logarithmic scale used to differentiate between less (negative) or more (positive) isoform expression relative to the tissue under comparison. The error bars represent ranges that are functions of the standard deviations of the ΔΔC_T _values (see Methods). **B) **Similar expression profiles for sodium channel isoforms were observed for lamina I/II spinal cord tissue taken from more mature Wistar rats (P25 to P30; n = 5).

In order to determine the specific sodium channel isoforms expressed within lamina I/II neurons we utilized qRT-PCR. Horizontal slices of the superficial-most dorsal horn (approximately 200 to 300 μm thick) were cut from the lumbar enlargement of Wistar rats (P6 to P9, as for electrophysiological experiments) to isolate predominantly lamina I/II tissue [23]. RNA was isolated followed by reverse transcription and real-time PCR using primers specific for individual sodium channel isoforms. Following normalization to an endogenous control (actin), isoform expression was normalized to Na_V_1.7 for illustrative purposes. As shown in the left panel of Figure 5A, the Na_V_1.2 and Na_V_1.3 isoforms were the most robustly expressed sodium channel isoforms within lamina I/II spinal cord. Positive controls demonstrated expression of glial (glial fibrillary acidic protein; GFAP) and neuronal (neuron-specific enolase; NSE) markers in lamina I/II spinal cord at higher levels than any of the sodium channel isoforms, and normalization to an alternate endogenous control gene (glyceraldehyde-3-phosphate dehydrogenase; GAPDH) yielded similar results to that shown in Figure 5A (data not shown). As negative controls, sodium channel isoforms known to be preferentially expressed in either skeletal muscle (Na_V_1.4), cardiac tissue (Na_V_1.5), or DRG neurons (Na_V_1.8 and Na_V_1.9) were examined and found to display negligible expression within the lamina I/II lumbar spinal cord. For the isoforms shown to be robustly expressed within lamina I/II tissue (Figure 5A, *left*), a more rigorous comparison was performed with normalization to control DRG tissue or the remainder of the spinal cord. Figure 5A (*right*) demonstrates that expression of the Na_V_1.2 and Na_V_1.3 isoforms is much greater (up to 30 fold) in lamina I/II tissue compared to either DRG or spinal cord lamina III - X samples, while Na_V_1.1 and Na_V_1.6 expression in lamina I/II tissue is less than in the control samples. The much greater expression (~10 to 1000 fold) of Na_V_1.7 (Figure 5A), Na_V_1.8, and Na_V_1.9 (data not shown) isoforms in DRG samples compared to lamina I/II verified that all sodium channel isoform primers were functional. To test whether the findings from young rats (P6 to P9) changed with development, qRT-PCR experiments were performed on samples from more mature (P25 to P30) rats. A similar predominant expression of the Na_V_1.2 and Na_V_1.3 isoforms in lamina I/II tissue was observed in the older rats, with a slight increase in the relative expression of Na_V_1.1 and Na_V_1.6 isoforms (Figure 5B).

The biophysical properties of sodium channel currents within lamina I/II neurons further support the qRT-PCR data showing predominant expression of a combination of Na_V_1.2 and Na_V_1.3 isoforms. Comparing our values to those reported for recombinant Na_V_1.2, Na_V_1.3 and Na_V_1.7 isoforms using similar recording protocols [24], the hyperpolarized voltage dependence of sodium channel activation and inactivation and the fast rates for onset of closed-state inactivation and recovery from inactivation in lamina I/II neurons closely align with values reported for recombinant Na_V_1.3 and Na_V_1.2 channels, respectively (Table 1).

**Table 1:** Comparisons of the biophysical properties of lamina I/II sodium channel currents to recombinant sodium channels

Na_V _Type	Activation V_0.5 _(mV)	Fast Inactivation V_0.5 _(mV)	Slow Inactivation V_0.5 _(mV)	Recovery from Inactivation τ at -80 mV (ms)	Development of Inactivation τ at -70 mV (ms)
DRG TTX-S Na_V _(Axotomized Rats)	-29	-72		34	156
**Lamina I/II Na_V_**	**-34**	**-69**	**-58**	**27**	**51**
Na_V_1.3	-26	-65		60	149
Na_V_1.2	-22	-61		18	33
Na_V_1.7	-26	-78		113	147

Bolded data are from the current study, while axotomized DRG, Na_V_1.3, Na_V_1.2, and Na_V_1.7 data are from [24].

### Physiological Implications of Sodium Channel Biophysical Properties

**Figure 6:**
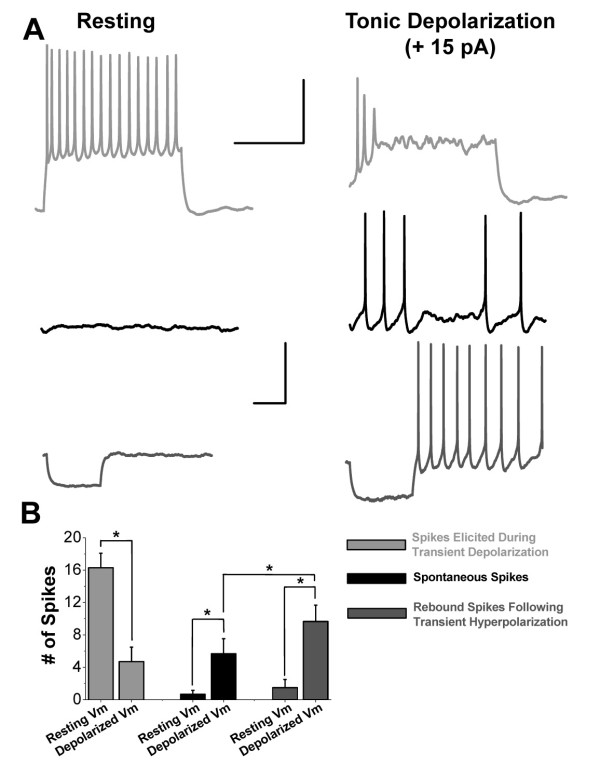
**Depolarization-induced changes in lamina I/II neuronal excitability**. Current-clamp recordings from intact tonic-spiking lamina I/II neurons in spinal cord slices from P6 to P9 Wistar rats. Traces and analyses were from an IV protocol consisting of 1 s transient hyperpolaring/depolarizing current injections between -20 and +75 pA at +5 pA increments. **A) ***Left*, representative voltage traces demonstrating a lamina II neuron's response to 1 s transient current injections of +40 pA (*top*), 0 pA (*middle*), and -20 pA (*bottom)*. The neuron had a resting membrane potential of approximately -62 mV. *Right*, in the presence of tonic depolarization (+15 pA), the same neuron had a resultant resting membrane potential of approximately -50 mV and altered electrical responses to 1 s transient current injections of + 25 pA (*top*), 0 pA (*middle*), and -20 pA (*bottom*). Scale bar x axes = 500 ms, y axes = 25 mV. Top scale bar corresponds to top traces, while bottom scale bar corresponds to middle and bottom traces. **B) **Bar graph demonstrating a significant (* p < 0.05, n = 6, Student's paired *t *test) reduction in average number of spikes (> 20 mV amplitude) elicited by 1 s transient depolarizations (46 +/- 5 pA, n = 6) for tonically depolarized neurons compared to the same neurons in their resting state (left grey bars). Significant (* p < 0.05, n = 6, Student's paired *t *test) increases in spontaneous activity (*middle *black bars) and rebound activity (*right *dark grey bars) over a 2 s period were observed for tonically depolarized neurons compared to the same neurons in their resting state. For tonically depolarized neurons, rebound spiking following transient -10 pA current injection was significantly (* p < 0.05, n = 6, Student's paired *t *test) greater than spontaneous spiking over the same 2 s period. Resting V_m _= -68 +/- 2 mV, n = 6; Depolarized V_m _= -52 +/- 2 mV, n = 6. LJP = 14.6 mV, corrected.

The observed overlap in the voltage dependence of sodium channel availability and activation (Figure 2D) could have significant implications for lamina I/II excitability. Current clamp recordings were utilized to explore the intrinsic excitability properties of lamina I/II neurons (intact in the spinal cord slice) from age-matched rats (P6 to P9). The initial resting membrane potential of lamina I/II neurons was measured to be -64 mV +/- 2 mV (n = 16), which lies within the "window current" region for the sodium channels expressed in these cells. As sodium channel slow inactivation will also be induced at the resting membrane potentials of lamina I/II neurons (Figure 4C), changes in membrane potential may have a dramatic impact on the transition of channels between their closed, activated, fast inactivated and slow inactivated states, which will depend on the combined kinetics of all processes involved. The input resistance of these intact young neurons was measured to be 1600 +/- 150 MΩ (n = 16), suggesting that small changes in membrane currents may have dramatic effects on excitability. We explored the intrinsic excitability of tonic firing [25] lamina I/II neurons at resting membrane potentials and found minimal spontaneous activity (0.4 +/- 0.2 Hz, n = 6) (Figure 6A* left *middle trace) and robust firing in response to small transient depolarizing current injection (Figure 6A* left *top trace). When the same neurons were tonically depolarized (through continuous positive current injection; +20 +/- 6 pA, n = 6) spontaneous spike activity was significantly increased (Figure 6A middle traces, 6B), potentially due to an increased activation of sodium channels. Further, subsequent transient depolarizations generated significantly fewer spikes while transient hyperpolarizations generated significantly more rebound spikes at tonically depolarized potentials compared to resting membrane potentials, potentially due to the onset and recovery from sodium channel inactivation, respectively (Figure 6B). In support of this hypothesis, transient hyperpolarizations were shown to actually increase spontaneous neuronal excitability (spike firing) at tonically depolarized resting potentials (Figure 6B). However, as no voltage-gated channels were blocked during the current clamp experiments, both voltage-gated potassium and calcium channels may also be involved in the observed effects of depolarization/hyperpolarization on membrane excitability.

## Discussion

### Functional Implications of Sodium Channel Biophysical Properties

Sodium channel currents within lamina I/II neurons are unique, with properties that diverge dramatically from sodium channel currents mediated by the Na_V_1.1, Na_V_1.6, Na_V_1.7, Na_V_1.8, and Na_V_1.9 isoforms expressed in nociceptive DRG neurons [26]. The relatively hyperpolarized voltage dependences of both activation and inactivation combined with fast closed-state inactivation observed for lamina I/II sodium currents predicts that these channels will be highly sensitive to changes in membrane potential. Both tonic and transient depolarizations will rapidly open and inactivate lamina I/II sodium channels, while fast recovery from inactivation kinetics will enable hyperpolarizations to induce a rapid repriming of lamina I/II sodium currents. Thus, relatively small GABAergic and glutamatergic synaptic inputs may have profound effects on excitability within these neurons. Rapid channel repriming may also enable lamina I/II neurons to fire at relatively high frequencies compared to peripheral nociceptors [18], although these properties also depend on interactions with voltage-gated potassium and calcium channel activity.

Prolonged depolarization can induce voltage-gated sodium channels into slow inactivated states that are distinct but not mutually exclusive from fast channel inactivation mechanisms [27,28]. It has been shown that tonic depolarization can cause adaptation of action potential firing within nociceptive DRG neurons by promoting sodium channel slow inactivation [29]. As observed for other TTX-sensitive channels [30-32], we find that lamina I/II neuron sodium channels have a more depolarized and gradual (less steep) voltage dependence of slow inactivation compared to fast inactivation. Because TTX-resistant sodium channel currents in DRG neurons have a more hyperpolarized voltage dependence of slow inactivation compared to fast inactivation and also reach complete (100%) slow inactivation at depolarized potentials (> -30 mV), it has been suggested that slow inactivation will be more prevalent for TTX-resistant sodium channels in response to physiological stimuli [29]. Although true for DRG neurons, the more hyperpolarized voltage dependence of slow inactivation for lamina I/II sodium channel currents (V_0.5SlowInactivation _= -62 mV) compared to other TTX-sensitive *and *TTX-resistant currents (V_0.5SlowInactivation _between -30 mV and -45 mV) [29-32] demonstrates that slow inactivation of TTX-sensitive sodium currents is especially relevant for lamina I/II physiology. For example, at -60 mV very few TTX-resistant sodium channels in DRG neurons are in the slow inactivated state [29] while ~46% of sodium channels in lamina I/II neurons are in the slow inactivated state (Figure 4). In this regard, we observed that tonic depolarization induced dramatic adaptation of action potential firing within lamina I/II neurons. Thus, pain therapeutics that specifically target the sodium channel slow inactivated state could have profound effects on lamina I/II neuronal excitability [18].

We predict that our detailed characterization of the biophysical properties of sodium channel currents in lamina I/II neurons will enable more accurate modeling of the changes in neuronal excitability that are produced by the alterations in membrane potentials, ionic gradients and synaptic inputs that occur during neuropathic pain states [33]. As for sodium channels in peripheral nociceptors [34], determining how pronociceptive substances modulate sodium channels in lamina I/II neurons will provide a greater understanding of the neuronal mechanisms that underlie pathophysiological hyperexcitability during chronic pain states.

### Molecular Composition of Lamina I/II Sodium Channel Currents

Our qRT-PCR results reveal high transcription levels of the Na_V_1.2 and Na_V_1.3 isoforms throughout development in lamina I/II spinal cord tissue from Wistar rats. As glial cells exhibit very low expression of voltage-gated sodium channels [16,35], we conclude that lamina I/II neurons predominantly express the Na_V_1.2 and Na_V_1.3 isoforms. Indeed, the biophysical properties of sodium channel currents within lamina I/II neurons most closely resemble the properties of these two isoforms (see Table 1). Of the other sodium channel isoforms expressed within lamina I/II, Na_V_1.1 has a more depolarized voltage dependence of both activation and inactivation [36], Na_V_1.6 has a faster onset of closed-state inactivation and recovery from inactivation [37,38], and Na_V_1.7 has a slower recovery from inactivation [24] when compared to sodium channel properties in lamina I/II neurons. However, it should be noted that neurons within lamina I/II spinal cord compose a heterogeneous population with varying intrinsic electrical properties [25]. Our electrophysiological recordings and qRT-PCR measurements represent averages from throughout lamina I/II and individual neuronal types may express unique combinations of sodium channel isoforms and properties. Future experiments could investigate this issue by combining sodium channel recordings with cell type-specific markers and single-cell RT-PCR experiments. Further, genetic knockdown experiments could be utilized to verify the specific contributions of the Na_V_1.2 and Na_V_1.3 isoforms to the biophysical properties of sodium channel currents within specific lamina I/II neurons.

Heterogeneity in the spinal cord also helps explain discrepancies between previous sodium channel labeling studies. It was originally shown that Na_V_1.3 spinal cord expression decreased during rat development, with minimal expression in the adult, while Na_V_1.2 was highly expressed throughout development, and Na_V_1.1 and Na_V_1.6 expression increased during development [39,40]. However, recent studies have demonstrated significant expression of Na_V_1.3 transcripts and protein in the adult spinal cord [35,41]. This apparent discrepancy may be partially due to differential expression of sodium channel isoforms between specific spinal cord lamina. *In situ *hybridization, RT-PCR, and immunohistochemical results have shown that the Na_V_1.1 and Na_V_1.6 isoforms are highly expressed throughout the adult rat spinal cord *except *in lamina II, while both Na_V_1.2 and Na_V_1.3 are highly expressed in the adult superficial dorsal horn, with Na_V_1.3 being specifically localized to lamina I/II [35]. At a functional level, it has been shown that intrinsic lamina I/II neuronal membrane properties that depend on sodium channel currents (rheobase, AP threshold, AP amplitude) do not change significantly between P6 to P10 and P21 to P25 [42]. In agreement with these recent findings, we observe that the predominant expression of Na_V_1.2 and Na_V_1.3 in lamina I/II is conserved between P6 to P9 and P25 to P30, with a slight increase in Na_V_1.1 and Na_V_1.6 in the older age range (Figure 5). This indicates that studying dorsal horn sodium channel currents using the ESI recording method from young (P6 to P9) rats is an appropriate model system for studying sodium channel function and modulation within nociceptive spinal cord processes.

### The Potential Roles of Na_V_1.3 in Nociceptive Signaling

We provide strong evidence that Na_V_1.3 channels are functionally expressed within lamina I/II neurons of naïve rats. It has been previously well-documented that Na_V_1.3 expression is upregulated within both nociceptive DRG and lamina I/II neurons during pathophysiological neuropathic pain signaling [3]. The increased expression of Na_V_1.3 following peripheral axotomy in DRG neurons is thought to underlie the acceleration of TTX-sensitive sodium current repriming observed in this peripheral neuropathic pain model [24]. Interestingly, the biophysical properties of DRG TTX-sensitive sodium channel currents in axotomized rats align very closely with lamina I/II sodium channel currents in naïve rats (except for the development of closed-state inactivation parameter; Table 1). In this regard, sodium channel currents expressed within lamina I/II neurons of naïve animals may be a suitable model system for studying pathophysiological TTX-sensitive Na_V_1.3 sodium currents without having to induce neuropathic pain states. Further, the functional expression of Na_V_1.3 in lamina I/II neurons of naïve rats suggest that this isoform may play important roles in both acute and chronic pain signaling mechanisms.

## Conclusions

Utilizing the space clamp advantages achieved through the ESI recording configuration [16], this study comprehensively characterizes the biophysical properties of sodium channel currents within lamina I/II neurons. Sodium channels within lamina I/II neurons generate rapidly repriming currents that activate and inactivate near resting membrane potentials, undergo relatively fast open-state and closed-state inactivation, and recover rapidly from inactivation. Recordings of neuronal excitability indicate that these sodium channel biophysical properties likely contribute to the somewhat surprising finding of increased excitability produced by transient hyperpolarizations during tonically depolarized states. A combination of molecular, biophysical and pharmacological evidence all support the conclusion that sodium channel currents within lamina I/II neurons are largely mediated by Na_V_1.2 and Na_V_1.3 isoforms, a highly divergent complement compared to the sodium channel isoforms previously shown to be expressed within peripheral nociceptors.

## Methods

### Ethical approval

All electrophysiological experiments involving rats and their care were performed in accordance with the recommendations of the Canadian Council on Animal Care and were according to the animal care regulations and policies of the University of British Columbia and the Hospital for Sick Children, Toronto.

### Spinal cord isolation

Male Wistar rats (P6 to P9; also P25 to P30 for qRT-PCR) were anaesthetized through intraperitoneal injection of Inactin (Sigma) or through inhalation of isoflurane. As previously described [18], the spinal cord was then rapidly dissected through ventral laminectomy and placed in an ice-cold protective sucrose solution containing (in mM): 50 sucrose, 92 NaCl, 15 D-Glucose, 26 NaHCO_3_, 5 KCl, 1.25 NaH2PO4, 0.5 CaCl_2_, 7 MgSO_4_, 1 kynurenic acid, and bubbled with 5% CO_2_/95% O_2_. For patch clamp electrophysiological recordings, transverse slices (300 to 400 μm) were cut using a vibratome, while horizontal slices of the superficial-most dorsal horn (approximately 200 to 300 μm) were cut for qRT-PCR experiments. Spinal cord lamina III - X tissue samples collected for qRT-PCR consisted of the remaining spinal cord after the first horizontal slice of the superficial dorsal horn was removed. Thoracic and lumbar DRGs were also collected during spinal cord dissections as an additional control for qRT-PCR experiments.

### qRT-PCR

Five superficial dorsal horn spinal cord samples from both P6 to P9 Wistar rats and P25 to P30 Wistar rats were used to determine relative gene expression of sodium channel isoforms using the 2^-ΔΔCT ^method for relative quantification [43]. All reagents were purchased from Applied Biosystems (Foster City, CA) unless otherwise noted. For reverse transcription, total RNA was extracted and DNAase treated from frozen spinal cord slices using RNAqueous^®^-4PCR reagents. cDNA was then transcribed according to the High Capacity cDNA Reverse Transcription Kit manual. A negative control (no reverse transcriptase) was carried out concurrently. For qRT-PCR, experiments were performed using the 7500 Real-Time PCR System (Applied Biosystems, Foster City, CA) with commercially available TagMan gene expression assays and primer/probe sets that were specific for individual sodium channel α subunits (Rn01638162_m1, Rn00680558_m1, Rn01485339_m1, Rn01461132_m1, Rn00689923_m1, Rn00668879_m1, Rn01532607_m1, Rn0154988_m1, Rn01403940_m1, Rn01445863_m1).

The Tagman amplicons span exon-exon junctions of the gene target and thus do not detect genomic DNA. Reactions were performed in 20 μL volumes containing 1 × Tagman Universal Master Mix, 1 × Tagman gene expression assay and cDNA template. The thermocycling protocol included an initial cycle at 50°C for 2 min and 95°C for 10 min, followed by 40 cycles of PCR at 95°C for 15 s and 60°C for 1 min. Each sample was run in experimental triplicate. Data were individually normalized to a passive internal control, ROX dye, followed by background subtraction. To normalize for the target template input, an endogenous control (β-Actin; ACTB 4352340E) was used concurrently, in experimental triplicates, for each sample. Individual isoform data were also compared to expression from DRG and spinal cord lamina III - × tissue samples collected from P25 to P30 Wistar rats using the Comparative C_T _Method as outlined in User Bulletin # 2 of Applied Biosystems.

### Electrophysiological recordings on lamina I/II spinal cord neurons

Transverse spinal cord slices were allowed to recover for 1 hour at 35°C in Ringer solution containing (in mM): 125 NaCl, 20 D-Glucose, 26 NaHCO_3_, 3 KCl, 1.25 NaH2PO4, 2 CaCl_2_, 1 MgCl_2_, 1 kynurenic acid, 0.1 picrotoxin, bubbled with 5% CO_2_/95% O_2_. The slice recovery chamber was then returned to room temperature (20 to 22°C) and all recordings were performed at this temperature. Cells were visualized using IR-DIC optics (Zeiss Axioskop 2 FS plus, Gottingen, Germany) and neurons from lamina I and the outer layer of lamina II were selected based upon their location relative to the substantia gelatinosa layer. Neurons were subject to patch clamp recording using borosilicate glass (BF150-86-10; Sutter Instruments, Navato, CA, USA) patch pipettes made from a Sutter P97 puller, with typical resistances of 3 to 6 MΩ (polished using Narishige MF-830 Microforge, Tokyo, Japan). Voltage clamp recordings of sodium channel currents in lamina I/II neurons were performed by pulling the neurons off the slice to enable adequate space clamp (ESI technique as in [16]). Neuron withdrawal from the slice took approximately 2 to 5 minutes with constant negative pressure applied to the recording pipette. All patch clamp recordings were performed using a Multiclamp 700B amplifier (Molecular Devices, Sunnyvale, CA, USA) connected to a personal computer running pClamp10 software through a Digidata 1440A Data Acquisition System (Molecular Devices). Neurons with peak inward sodium currents (I_peak_) less than 100 pA or inward leak currents greater than 50 pA (I_leak _= -29 +/- 3 pA, n = 39; at V_hold _= -90 mV) were discarded before analysis. Neurons typically had access resistances below 20 MΩ and capacitance and series resistance compensation of 50% to 70%, although compensation was removed in some cases (as done in [16]) with no change in channel kinetics or voltage dependence. Leak subtraction was performed off-line using pClamp10 software (Molecular Devices). As neurons do not remain viable at prolonged highly hyperpolarized holding potentials (e.g. V_hold _= -120 mV), the time course (Figure 1) and IV (Figure 2A,B) protocols contained a 200 ms prepulse to -120 mV to remove channel fast inactivation, with V_hold _= -80 mV.

The external recording solution consisted of a modified TEA-Ringer solution containing (in mM): 95 NaCl, 20 TEACl, 11 D-Glucose, 25 NaHCO_3_, 5.6 KCl, 1 NaH_2_PO_4_, 0.1 CaCl_2_, 5 MgCl_2_, 1 kynurenic acid, 0.1 picrotoxin, while the internal patch pipette solution contained (in mM): 140 CsCl, 5.8 NaCl, 1 MgCl_2_, 3 EGTA, 10 HEPES, 4 MgATP, 0.5 Na_2_GTP, adjusted to pH 7.3 with NaOH and 290 mOsm with D-mannitol (if necessary). A calculated liquid junction potential (LJP) of 4.2 mV remained uncorrected. Voltage clamp recordings were digitized at 50 KHz and low-pass filtered at 2.4 kHz. Current clamp recordings were performed on intact lamina I/II neurons using the recovery Ringer solution, while the internal patch pipette solution contained (in mM): 140 KGluconate, 4 NaCl, 10 HEPES, 1 EGTA, 0.5 MgCl_2_, 4 MgATP, 0.5 Na_2_GTP, adjusted to pH 7.2 with 5 M KOH and to 290 mOsm with D-Mannitol (if necessary). Only tonic firing neurons [25] were selected for current-clamp experiments investigating the effects of membrane potential on spike firing, while resting membrane potentials were collected for recordings from all neuronal firing pattern types. Nine of 16 recorded neurons were found to be tonic spiking. For current-clamp recordings, a calculated LJP of 14.6 mV was corrected offline and recordings were digitized at 50 kHz and low-pass filtered at 10 kHz.

### Compounds and perfusion

Unless otherwise indicated, all compounds were ordered from Sigma. A closed perfusion system (10 mL) was used for spinal cord slice recordings, with a flow rate of between 2 and 4 mL/min.

### Data analysis

Figures and data fittings utilized Microcal Origin 8.5 (Northampton, MA, USA). Current-voltage relationships were fitted with the modified Boltzmann equation: *I = *[G_max_*(*V_m_-E_rev_*)]/[1*+*exp((*V_m_*-*V_0.5a_*)*/k_a_*)], where *V_m _*is the test potential, *V_0.5a _*is the half-activation potential, *E_rev _*is the extrapolated reversal potential, *G_max _*is the maximum slope conductance, and *k_a _*reflects the slope of the activation curve. The voltage dependence of both fast and slow inactivation were fit with standard Boltzmann equations. The onset of inactivation and recovery from inactivation curves were both fit with monoexponential equations, as this provided the optimal fit for the majority of neurons. Data from concentration-dependent response studies were fit with a Hill equation, as described in [18]. All data are given as means +/- standard error of the mean.

## Abbreviations

DRG: dorsal root ganglia; ESI: entire soma isolation; GAPDH: glyceraldehyde-3-phosphate dehydrogenase; GFAP: glial fibrillary acidic; IC_50 _= concentration that produces 50% inhibition of the control response; LJP: liquid junction potential; NSE: neuron-specific enolase; qRT-PCR: quantitative real-time RT-PCR; TTX: tetrodotoxin.

## Competing interests

Zalicus Pharmaceuticals Ltd. is a subsidiary of Zalicus Inc. JM, PLS, ET, and TPS all hold shares and/or options in Zalicus Inc.

## Authors' contributions

Experiments were performed under the direction of TPS, ET and MWS in their laboratories at the University of British Columbia, Zalicus Pharmaceuticals Ltd. and the Hospital for Sick Children, respectively. All authors contributed to the conception and design of experiments and the drafting and revising of the manuscript. MEH, JM and PLS contributed to the collection, analysis, and interpretation of data. Specifically, MEH performed all electrophysiology experiments, PLS collected DRG and spinal cord samples for qRT-PCR, and JM performed qRT-PCR experiments. All authors approved the final version of this manuscript.
